# Laboratory diagnosis and susceptibility profile of *Helicobacter pylori *infection in the Philippines

**DOI:** 10.1186/1476-0711-3-25

**Published:** 2004-11-16

**Authors:** Raul V Destura, Eternity D Labio, Leah J Barrett, Cirle S Alcantara, Venancio I Gloria, Ma Lourdes O Daez, Richard L Guerrant

**Affiliations:** 1 Center for Global Health, Division of Infectious Disease and International Health, University of Virginia, Charlottesville, Virginia, USA; 2 Section of Infectious Diseases, Philippine General Hospital, Manila, Philippines; 3 Section of Gastroenterology, Department of Medicine, Philippine General Hospital, Manila, Philippines; 4 National Institute of Health-University of the Philippines, Manila, Philippines

## Abstract

**Background:**

*Helicobacter pylori *diagnosis and susceptibility profile directs the applicability of recommended treatment regimens in our setting. To our knowledge, there is no published data on the culture and local susceptibility pattern of *Helicobacter pylori *in the Philippines.

**Methods:**

52 dyspeptic adult patients undergoing endoscopy from the Outpatient Gastroenterology clinic of the University of the Philippines-Philippine General Hospital underwent multiple gastric biopsy and specimens were submitted for gram stain, culture, antimicrobial sensitivity testing, rapid urease test and histology. Antimicrobial susceptibility testing was done by Epsilometer testing (Etest) method against metronidazole, clarithromycin, amoxicillin, and tetracycline.

**Results:**

Sixty percent (60%) of the study population was positive for *H. pylori *infection (mean age of 44 years ± 13), 70% were males. *H. pylori *culture showed a sensitivity of 45% (95% CI [29.5–62.1]), specificity of 98% (95%CI [81.5–100%]), positive likelihood ratio of 19.93 (95% CI [1.254–317.04]) and a negative likelihood ratio of 0.56 (95% CI [0.406–0.772]). All *H. pylori *strains isolated were sensitive to metronidazole, clarithromycin, amoxicillin and tetracycline.

**Conclusion:**

Knowledge of the antibiotic susceptibility patterns in our setting allows us to be more cautious in the choice of first-line agents. Information on antibiotic susceptibility profile plays an important role in empiric antibiotic treatment and management of refractive cases.

## Background

*Helicobacter pylori *is a gram-negative bacterium that colonizes the gastric mucosa of more than half of the world's population [1,2] Since its isolation in 1982, the association between *H. pylori *infection and the subsequent development of chronic active gastritis, peptic ulcer disease, gastric cell carcinoma and B cell MALT lymphoma has been well established[3]. The principal reservoir of infection is the human stomach and transmission has been epidemiologically linked to person to person contact [4]. The prevalence of infection is greater in developing countries and is influenced by socioeconomic conditions, ethnic background and age[5,6] In the Philippines, there is scarcity of published data regarding the epidemiology of this bacterium. Locally unpublished reports revealed a prevalence of 5.6% seropositivity rate in children and 60% among 136 adult Filipino patients with dyspepsia using the Clotest^® ^(Cabahug et. al. 2003 and Caballero et al., 1997, *unpublished data*). A lower prevalence rate of 42% was reported by Daez et. al. in 2002 (*unpublished data*) among 375 patients undergoing endoscopy at the Philippine General Hospital utilizing the rapid urease test and histopathology.

Microbiological isolation of the organism is the theoretical gold standard for the detection of *H. pylori *infections. However, isolation of the organism by culture has been highly variable. Success rates depend on the technical expertise of the microbiology laboratory, ranging from 30% to 73%[7,8]. Failure to detect the organism may be due to sampling error, inappropriate transport or culture media and insufficient incubation period.

In clinical practice, gastric biopsy with culture is not routinely performed due to the availability of more rapid diagnostic tests in the detection of *H. pylori *such as urease broth tests, urea breath tests, serologic methods and stool antigen detection. However, the increasing prevalence of resistant strains makes culture and antibiotic sensitivity testing valuable to determine alternative treatment regimens after failure of initial eradication regimen.

In the Philippines, due to methodological difficulties in isolating the organism, detection of the organism by culture methods has not been popular. Realizing the increasing prevalence of antimicrobial resistance in other countries and its potential negative impact on the efficacy of many treatment eradication regimens, it is important in clinical practice to determine the prevailing local antibiotic susceptibility patterns when choosing appropriate eradication regimens for *H. pylori *infections in the empiric setting.

This study aims to evaluate the use of culture in the diagnosis of *H. pylori *infection among patients with dyspepsia, to determine the sensitivity and specificity of culture technique in the detection of *H. pylori *infection, and to determine the antibiotic susceptibility patterns of *H. pylori *organisms isolated by culture among Filipino patients.

## Methods

This is a prospective, cross-sectional study involving adult patients with dyspepsia, who had independently been determined to have clinical indications for an endoscopy at the out-patient gastrointestinal clinic of the Philippine General Hospital, a tertiary training university hospital in Manila. Eligible patients were enrolled in the study after informed consent to undergo the required diagnostic testing of endoscopy samples.

Patients were excluded if they were less than 18 years old, had a history of proton pump inhibitors (PPI) intake within 2 weeks, H2 antagonists within 1 week and antibiotic intake within 1 month prior to inclusion in the study.

The nature and purpose of the study were discussed with the patient until fully understood. All patients with dyspepsia undergoing endoscopy who fulfilled the inclusion criteria had a complete history and physical examination. Data were obtained using a data collection form. Participants underwent upper gut endoscopy as clinically indicated. Pre-procedure preparations for Esophagogastroduodenoscopy were performed according to standard methods. Biopsy of gastric tissue were collected from the antrum and body of the stomach and specimens were sent for (1) histopathologic study, (2) gram staining, (3) culture and sensitivity and (4) rapid urease broth test. Those interpreting results of the above diagnostic tests were blinded.

### Case Definition

A patient with *Helicobacter pylori *infection was defined as those patients independently assessed by their attending physician based on clinical symptoms and a positive test for any of the two diagnostic tests (histology and rapid urease test). In the evaluation of the diagnostic performance of *H. pylori *culture, the above clinico-laboratory case definition were used as the comparator reference standard.

### Description of the Diagnostic Tests

#### Gram stain and Culture

Two pieces of gastric tissue were obtained and placed in 0.2 mL sterile saline and transported to the microbiology laboratory for processing. The biopsy specimen was placed in a sterile petri dish and minced with 2 sterile scalpel blades. Specimens were inoculated in both 7% Horse Blood + Brain Heart Infusion Agar (HBAP) and Brucella Blood Agar with Skirrow's supplement (5% defibrinated Sheep's Blood with Trimethoprim (5 mg/L), Vancomycin 10 mg/L and Polymixin B (2500 units/L))[9] Plates were incubated at 37°C for 7–10 days in a microaerophilic incubation environment and examined every other day (Pack-Microaero, Mitsubishi gas co., Japan). *H. pylori *colonies are typically small, flat and translucent to grey. On days 4 to 5, all plates with no characteristic colonies, were subcultured to a fresh HBAP to promote growth of slow or fastidious strains and incubated for an additional 3 to 5 days. Plates were examined until the tenth day before reporting a negative growth. Suspected *H. pylori *colonies were tested for urease, oxidase and catalase production. A modified gram stain was performed on a methanol-fixed smear using crystal violet for 1 minute followed by a water wash and then a safranin counter stain for 30 minutes prior to a final washing with tap water The smear was air-dried and examined under oil immersion.

The presence of *H. pylori *is confirmed by the presence of a gram negative curved bacilli and a positive test for urease, oxidase and catalase production. Five *H. pylori *isolates that survived the shipping and handling were verified at University of Virginia Center for Studies of Diseases Due to *H. pylori*.

### Sensitivity Study

Epsilometer test (Etest, AB Biodisk, USA) was used to determine the minimal inhibitory concentrations (MIC). MIC values were read as the intercept of the elliptical zone of inhibition with the graded strip for the Etest. Strains were considered resistant when the MIC was >8 g/ml for metronidazole, >1 g/ml for clarithromycin and >0.5 g/ml for amoxicillin. These breakpoints were used based on the recommendations from the National Committee for Clinical Laboratory Standards (NCCLS) and a large clinical trial [10,11]. For tetracycline, resistance was determined at an MIC of >2 μg/ml based on a previous publications[12,13]. Sensitivity results were compared with a standard susceptible strain of *H. pylori *(NCTC # 12822) and the University of Virginia Center for Studies of Diseases Due to *H. pylori *metronidazole resistant culture strain #Cp 2124 and clarithromycin resistant strain #Cp 5535. In the absence of a resistant control for amoxicillin and tetracycline, susceptibility breakpoints set by NCCLS and large clinical trials were used[10,11,13].

#### Rapid Urease Test

The Rapid Urease Test (RUT) was performed by placing 0.5 ml of 8% (weight/vol) unbuffered urea in distilled water (pH 6.8) in a clear 0.7 ml Eppendorf tube, to which one drop of 1% phenol red (free acid) suspension was added. The urea solutions was stored at 4°C and prepared on the day of use to ensure color stability. Two gastric biopsy specimens from the antrum and body were placed in the tube. A positive test was indicated by a rapid color change of the media surrounding the biopsy from yellow to magenta followed by a rapid generalized color change throughout the media. A negative result was indicated when there was no change in color appreciated after 2 hours of observation.

#### Histopathologic Examination

Specimens were sent to the pathology lab and gastric tissues were fixed and stained with Giemsa and Hematoxylin-Eosin dye. A specimen was read as positive if curve bacilli organisms were seen on microscopy. Pathologists were blinded to the results of the other diagnostic tests.

### Statistical Analysis

Demographic data was described using rates and percentages for categorical variables. For continuous variables, means and standard deviations were used. Measures of accuracy for *H. pylori *culture were expressed as sensitivity and specificity rates, positive and negative predictive values and likelihood ratios with a 95% confidence interval.

## Results

### Patient Characteristics

Among 52 patients with dyspepsia, 31 (60%) were positive for *H. pylori *infection based on the pre-defined case definition. The mean age for *H. pylori *infected individuals was 44 years ± 13. Seventy percent were males with a male:female ratio of 2:1. The majority of infected patient were married (80%), and had reached only up to the secondary level of education (70%). Fifty-five percent were unemployed. Seventy-five percent of infected patients had access to piped water. No significant differences among the demographic characteristics of *H. pylori *positive and negative cases were observed.

### Laboratory Diagnosis of H. pylori

**Figure 1:**
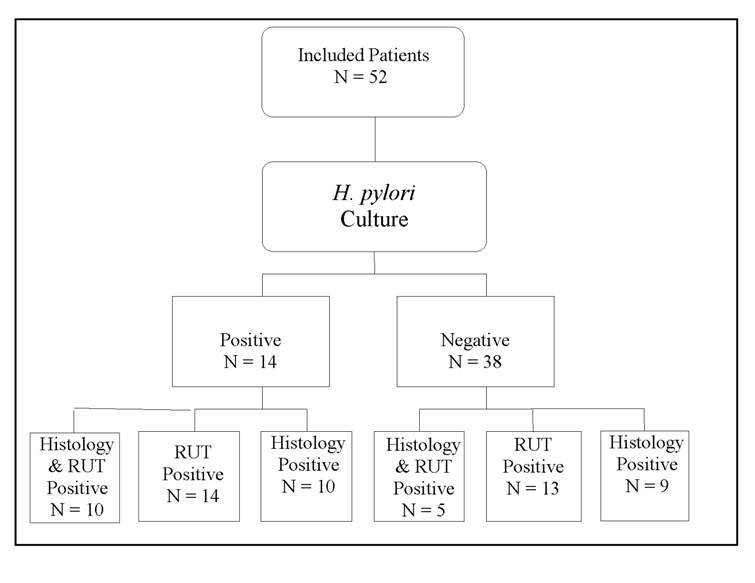
**Comparative positivity of diagnostic tests: RUT (rapid urease test) and histology to *H. pylori *culture**. All patients underwent biopsy, RUT and culture studies. Among the 31 clinico-laboratory defined cases, 14 were culture positive. Ten of the *H. pylori *culture positive cases were also positive for both histology and RUT. All 14 culture positive isolates tested positive for RUT while only 10 were positive for histology.

All included patients underwent the 3 diagnostic tests for the diagnosis of *H. pylori *infection (histopathology, rapid urease test (RUT) and culture) (figure 1). Fourteen of the 52 patients grew *H. pylori *on culture. Ten of the 14 positive culture samples were also positive for both histology and RUT, 14 and 10 were positive for RUT and histology alone, respectively. To validate the accuracy rate of *H. pylori *culture, results of the culture studies were compared with clinically defined cases of *H. pylori *infection, in this case patients who presents with abdominal symptoms and positive for at least one of the two diagnostic tests, histopathology and RUT. *H. pylori *culture showed a sensitivity of 45% (95% CI [29.5–62.1%]), specificity of 98% (95%CI [81.5–99.8%]), positive likelihood ratio of 19.93 (95% CI [1.254–317.04]) and a negative likelihood ratio of 0.56 (95% CI [0.406–0.772]). The positive predictive value was 97% (95% CI [74.7–99.7%])) and the negative predictive value was 55% (95% CI [39.8–69.7%]).

A total of 14 *H. pylori *organisms were isolated from 52 clinical specimens. The mean number of incubation time was 3.8 days ± 1 day. All isolates grew on primary plates. All isolates were highly sensitive to amoxicillin (mean MIC of 0.016 ug/ml by Etest)), tetracycline (mean MIC of 0.164 ug/ml SD ± 0.16 SD by Etest), metronidazole (mean MIC of 0.061 ug/ml SD ± 0.04 by Etest) and clarithromycin (mean MIC of 0.016 SD ± 0 by Etest) (table 1).

## Discussion

This pilot study reported an *H. pylori *culture sensitivity rate of 45% and a specificity rate of 98–100% which are comparable to those reported in other countries[8,14-17]. High positive predictive values coupled with an intermediate to high likelihood ratio demonstrates that gastric tissue culture is highly specific, making it a useful confirmatory test in the diagnosis of *H. pylori *infection. Its low sensitivity is acceptable since this method is not recommended as a screening test. This study also supported previous studies that the rapid urease test whether in a gel or liquid preparation is a highly sensitive tool which qualifies as a good screening test among suspected *H. pylori *infected individuals[13,18,19].

Antibiotic resistance has increasingly been recognized as a major cause of treatment failure for *H. pylori *infection. Primary antimicrobial resistance against clarithromycin and metronidazole is now commonplace in several countries[2,20-26]. Regional variations in susceptibility and resistance patterns may be ascribed to differences in local antibiotic prescription practices, antibiotic usage in the community and mass eradication programs for *H. pylori *infection as part of gastric cancer prevention strategies. These factors may well be expected to influence success of eradication therapy [27-29].

All 14 strains isolated showed sensitivity to all the first line antibiotics namely metronidazole, amoxicillin, clarithromycin and tetracycline. No resistant strains were isolated based on the Etest method. Susceptibility patterns in Europe and the United States revealed that the highest resistance is to metronidazole ranging from 33.1% to 36.9%. Clarithromycin resistance was observed to be 10% in both areas. In contrast, Japanese data showed that clarithromycin resistance was 29% closely followed by metronidazole at 24%. Amoxicillin resistance remained low at 0–1.4% in all three geographic locations[3,21,23]. While susceptibility studies were done on large numbers of isolates in foreign data, this local study comprises one of the pioneering attempts, to determine the antibiotic susceptibility pattern of *H. pylori *infection in the Philippines.

Potential reasons for the absence of resistant *H. pylori *strains in our pilot study point to the type of population enrolled. Based on the selection criteria, these patients had no exposure to previous antibiotic nor had previous *H. pylori *eradication treatment, a strong risk factor for the development of acquired resistance. Another possible explanation for the low resistance of *H. pylori *isolates as compared to other Asian countries, is the difficulty procuring antibiotics due to their restrictive cost. In this study, 70% of *H. pylori *positive patients were unemployed with average incomes below the poverty level, defined as income below the annual per capita poverty threshold of PHP 18,000.00 for the National Capital Region of the Philippines (2001 Philippine Health Situation, Department of Health, Manila, Philippines, ). A study of the World Health Organization's Programme for appropriate Health Care Technology (ATH) has shown a correlation between the occurrence of multi-resistant bacteria and antibiotic consumption patterns. The Philippines has the highest percentage in 1983 of antibiotic utilization among countries (including USA, Japan, United Kingdom) surveyed (>25%). However, majority of the people whether rich or poor allot minimum expenses for medical care at 2.7% and 1.2%, respectively[30]. Such paradox in resistance patterns may well be explained by the capacity of these patients to actually afford the prescribed duration of antibiotic therapy. While many Filipinos may be (mis-) guided on the appropriate choice of antibiotic therapy by media or product representatives, the cost of these drugs still limits the access to these largely economically disadvantaged group. Although the presence of primary resistance of *H. pylori *has been well documented in other studies, the absence of primary resistance in our results may also be an underestimate of the true prevalence of *H. pylori *resistance because of the smaller sample size compared with published literature[2,20,25,26]. Only with continued surveillance of susceptibility patterns and a larger sample size of isolates will provide a more substantial answer to the issue of resistance of *H. pylori *in the Philippines.

Knowledge of the antibiotic susceptibility patterns in our setting allows us to be more cautious in the choice of first-line agents. The use of culture technique in the diagnosis of *H. pylori *infection approximates that in published literature abroad. In the absence of standard disk diffusion zone sizes for regimens used in *H. pylori *eradication regimen except for metronidazole, further establishment of the susceptibility pattern of locally occurring isolates by comparing zone size breakpoints with Etest, agar dilution method and as well as molecular genotyping of resistant strains will be the future direction of this pilot study.

## Conclusions

While the use of culture is not an ideal test for the rapid diagnosis of *H. pylori *infection, information on antibiotic susceptibility profile plays an important role in empiric antibiotic treatment and management of refractive cases

## Authors' contributions

RVD, LJB, CSA contributed in the microbiologic isolation of *H. pylori*, manuscript writing and editing

EDL, MOD, VD contributed in the specimen processing of biopsy samples, manuscript writing and editing

RLG contributed in the research design planning, manuscript content and final editing

**Table 1:** Sensitivity study *Helicobacter pylori *isolates*

**ISOLATE NO.**	**AMOXICILLIN**	**TETRACYCLINE**	**METRONIDAZOLE**	**CLARITHROMYCIN**
	
	*Etest Resistance Breakpoints			
	
	**> 0.5 ug/ml**	**> 2 ug/ml**	**> 8 ug/ml**	**> 1 ug/ml**
1	0.016	0.032	0.016	0.016
2	0.016	0.19	0.125	0.016
3	0.016	0.016	0.016	0.016
4	0.016	0.38	0.064	0.016
5	0.016	0.38	0.064	0.016
6	0.016	0.19	0.125	0.016
7	0.016	0.032	0.016	0.016
8	0.016	0.38	0.016	0.016
9	0.016	0.032	0.064	0.016
10	0.016	0.19	0.016	0.016
11	0.016	0.38	0.016	0.016
12	0.016	0.032	0.125	0.016
13	0.016	0.032	0.064	0.016
14	0.016	0.032	0.125	0.016
Strain # CP2124^§^	0.015	0.015	33	0.015
Strain # CP5535^§^	0.015	0.125	1.5	257

* all Etest studies done on the Philippine isolates were in the sensitive ranges

^§^UVa (University of Virginia) Metronidazole resistant isolate

^¥^UVa Clarithromycin resistant isolate
